# Factors affecting hospital length of stay and hospital charges associated with road traffic-related injuries in Iran

**DOI:** 10.1186/1472-6963-13-281

**Published:** 2013-07-22

**Authors:** Hassan Haghparast-Bidgoli, Soheil Saadat, Lennart Bogg, Mohammad Hossein Yarmohammadian, Marie Hasselberg

**Affiliations:** 1 Division of Global Health, Department of Public Health Sciences, Karolinska Institute, Stockholm, Sweden; 2 University College London, Institute for Global Health, London, UK; 3 Health Management and Economics Research Centre, Faculty of Management and Informatics, Isfahan University of Medical Sciences, Isfahan, Iran; 4 Sina Trauma and Surgery Research Center, Tehran University of Medical Sciences, Tehran, Iran; 5 School of Health, Care and Social Welfare, Mälardalen University, Västerås, Sweden

## Abstract

**Background:**

Road traffic injuries (RTIs) are a substantial cause of mortality and disability globally. There is little published information regarding healthcare resource utilization following RTIs, especially in low and middle-income countries (LMICs). The aim of this study was to assess total hospital charges and length of stay (LOS) associated with RTIs in Iran and to explore the association with patients’ socio-demographic characteristics, insurance status and injury-related factors (e.g. type of road users and safety equipment).

**Method:**

The study was based on the Iranian National Trauma Registry Database (INTRD), which includes data from 14 general hospitals in eight major cities in Iran, for the years 2000 to 2004. 8,356 patients with RTI admitted to the hospitals were included in the current study. The variables extracted for the analysis included total hospital charges and length of stay, age, gender, socio-economic and insurance status, injury characteristics, medical outcome and use of safety equipment among the patients. Univariable analysis using non-parametric methods and multivariable regression analysis were performed to identify the factors associated with total hospital charges and LOS.

**Results:**

The mean hospital charges for the patients were 1,115,819 IRR (SD=1,831,647 IRR, US$128 ± US$210). The mean LOS for the patients was 6.8 (SD =8 days). Older age, being a bicycle rider, higher injury severity and longer LOS were associated with higher hospital charges. Longer LOS was associated with being male, having lower education level, having a medical insurance, being pedestrian or motorcyclist, being a blue-collar worker and having more severe injuries. The reported use of safety equipment was very low and did not have significant effect on the hospital charges and LOS.

**Conclusion:**

The study demonstrated that the hospital charges and LOS associated with RTI varied by age, gender, socio-economic status, insurance status, injury characteristics and health outcomes of the patients. The results of the study provide information that can be of importance in the planning and design of road traffic injury control strategies.

## Background

Road traffic injuries (RTIs) are a major cause of morbidity and mortality globally, with a disproportionate number occurring in low and middle-income countries (LMICs) [1]. Apart from the human suffering, RTIs are also responsible for economic losses of approximately 1–1.5% of the annual gross national product (GNP) in LMICs [1].

Iran has one of the highest RTI death rates in the world [2,3]. RTIs are considered to be the second cause of mortality in the country, after cardiovascular diseases [2]. RTIs pose a significant burden on the health care sector as almost 0.8 million people (1.1% of population) seek hospital care as a result of RTI annually [2]. Moreover, since traffic-related mortality and morbidity in Iran occur most commonly among young adults, the impact of RTIs (permanent disability and premature death) is huge both in terms of human suffering and economic consequences for families and for the society [2,4]. RTIs constitute the leading burden of disease in the country, with the highest number of Disability-Adjusted Life Years (DALYs) accrued for the population [2,4]. The annual cost of road traffic crashes in Iran is estimated to be approximately US$ 2 billion (about 2% of GNP) [4,5].

The costs and resource burden of traumas have been documented in high-income countries (HICs) [6-10], but few studies have been published from LMICs [11,12], particularly in relation to RTIs. In Iran, where information on the outcomes of RTI has been well documented in previous studies [13-15], there is little data reported on trauma care costs and resource utilization of RTIs. In particular, there are no published analyses of factors affecting resource utilization by RTI victims. Such information is valuable for policy makers in Iran and in other LMICs with similar context in order to prioritize injury prevention and trauma care interventions.

Previous studies in HICs, have identified factors such as age, gender, injury severity, hospital mortality, insurance type and hospital location as predictors of hospital charges and length of stay for injuries [6-10]. Studies have also shown that the use of safety equipment (helmet and seat-belt) improves medical outcomes and reduces health care resource utilization [16-19].

The current study aimed to assess hospital charges and LOS associated with RTIs in Iran and to explore the relationship with socio-demographic characteristics, insurance status and injury-related factors (e.g. type of road users and safety equipment) of the patients.

## Method

### Setting

Iran, a low-middle income country, is located in the Eastern Mediterranean Region and covers 1,648,000 sq km [20]. The country has over 70 million inhabitants, of whom 69% live in urban areas [21]. Vehicle ownership is significantly higher in Iran than in other countries with similar income levels [2]. Based on estimates from the 2006 Census, Iran had almost six million cars and over five million motorized two-wheeler vehicles [2]. With the growing pattern of urbanization and motorization during the past few years, there has been a rapid increase in traffic-related injury mortality and morbidity [3]. The RTI rate nearly quadrupled in just eight years, from 109.7 to 400.6 per 100 000 population between 1997 and 2005 [3]. Such rapid increase in RTIs has imposed great pressures on the health sector, insurance companies and patients and their families [4]. At the time of conducting this study, medical charges of RTI victims were covered for those with medical insurance of any kind (except for pre-hospital care provided by Emergency Medical Service or EMS, which was free of charge for all patients). The four major public insurance systems in Iran are; a) The Medical Service Insurance Organization; provides coverage for around 40% of population mainly government employees, rural households, the self-employed, and others such as students. It is largely financed through general tax revenues. b) The Social Security Organization; provides a range of benefits such as pensions, unemployment benefit, disability benefit and health insurance for around 30% of population, mainly workers in the non-governmental sector including self-employed and voluntary contributors, and contractual workers from the public sector. It is funded by a payroll tax shared by employer and employee and provides health services to its members either directly through its own hospitals and health centres or indirectly via contracts with other providers. c) The Armed Forces Medical Services Organization; covers about 5% of population included the members of the army, police and other armed forces and their dependents. It provides services mostly through contracting directly through its own facilities or via contracts with other providers. d) The Imam Khomeini Relief Committee; covers around 8% of population who cannot afford to pay for insurance. It is financed by charity and the government and provides services through contracts with different providers [22-25]. In addition there are other small semi-public companies which mainly provide complementary insurance policies, covering almost 5% of the middle-class population [23]. According to a newly approved law, which came into force in 2008, all hospital care for RTI victims is now free of charge [26]. The new law was implemented after our data was collected.

### Data source and variables

This study utilized the Iranian National Trauma Registry Database (INTRD). The database includes data from 14 general hospitals affiliated to the Medical Universities in eight major cities in Iran (Tehran, for 13 months; Mashhad and Ahwaz for 7 months; and Shiraz, Tabriz, Qom, Kermanshah, and Babul for 4 months). The data was collected between 2000 and 2004 by trained physicians using a validated questionnaire, in accordance with the American College of Surgeons National Trauma Registry System and the National Trauma Data Bank. During the data collection period, a group of trained physicians visited trauma patients after the first 24-hours of their admission to the hospital (at emergency department or inpatient wards) and completed the questionnaires (i.e.: the database does not cover out-patients).

A total of 17,753 trauma patients were recruited, among them 8,356 patients with road traffic-related injuries. These patients were included in the current study. The data gathered included socio-demographic characteristics of the patients (including age, sex, occupation and education), pre-hospital care (including transportation type and duration and care provided), type and mechanism of injury, coded according to the International Classification of Diseases, 10th revision (ICD-10) ^a^ , Glasgow Coma Scale (GCS) and vital signs at the time of entering the emergency department, Injury Severity Score (ISS), diagnostic and therapeutic procedures, co-existing injuries, length of stay (LOS) in hospital and intensive care unit, patient’s outcome (including discharge with recovery, death, short-term or long-term disabilities), total hospital charges for each patient and insurance type. Information such as socio-demographic, using safety equipment, insurance type were collected through interview with patients (or persons accompanying patients in case if the patient was too ill to report this information) and diagnostic information, LOS and the hospital charges (Iranian Rial-IRR) obtained from the patient’s medical and accounting records.

INTRD have mainly been used in epidemiological studies on various types of trauma [27-29]. This is the first study using this database to investigate total hospital charges and LOS related to RTIs and factors associated with them. Total hospital charges and LOS have been used, as measures for resource utilization, by previous trauma studies [6,7,30,31]. Total hospital charges are the amount billed by the hospitals for the services and do not reflect the actual medical costs for the injury, because of the governmental subsidies for hospital services and medicines and also discounts and exemptions for some patients given by the hospitals. The bill is issued before the patient discharge and comprises costs of all services the patient received (included items such as, hostel costs, diagnostic services, medicines, consultation fees, surgery,…) and included both patient and insurance company shares (if insured) of payments. Hospital charges were adjusted for inflation and converted to 2004 IRR using the consumer price index for health care in Iran from 2000 to 2004.

Moreover, the following information was extracted from the database: 1) socio-demographic status of patients; including age, sex, education, occupation and insurance status, 2) type of road users including pedestrians, car passengers or drivers, motorcycle riders, bicycle riders and others, 3) injury severity measures including GCS and ISS, 4) patient outcome measured by death in hospital and 5) use of safety equipment including helmet and seat-belt.

### Data analysis

Initially, the mean and median LOS and total hospital charges were compared in sub-categories of patients’ demographic and injury characteristics by using Mann Whitney U test (for dichotomous variables such as sex and insurance status) and Kruskal-Wallis test (for categorical variables such as education level, ISS, GCS, road user types, age group and occupation). Chi-Square and Mann–Whitney tests were used to compare the proportion of (in hospital) deaths and mean and median total hospital charges/LOS, respectively, among people who used safety equipment (helmet/seat-belt) and those who did not use safety equipment. A P-value of <0.05 was considered statistically significant.

Furthermore, multivariable regression analyses of hospital charges and LOS were conducted in order to identify the key predictors. Due to the skewed distribution of total hospital charges and LOS (outcome variables), they were log transformed. Multivariable regression was conducted separately for total hospital charges and LOS as dependent variables and age, sex, education, occupation, insurance status, ISS, GCS, road user types and death in hospital (and LOS for the model in which hospital charges was considered as dependent variable) as independent variables. A full model including all independent variables was initially estimated and then using a backward stepwise selection procedure, a final model including only those variables that had a statistically significant association with hospital charges or LOS was developed. The data were analyzed using SPSS software (version 13.0).

The study was approved by the Ethical Committee of the Sina Trauma and Surgery Research Center (STSRC). Informed consent was provided from all patients before they were enrolled in the study.

## Results

### Socio-demographics and injury characteristics of the study population

RTIs comprised nearly 50% (8,356 cases) of all traumatic patients admitted to the studied hospitals. The results showed that 78% of the patients were male (male–female ratio, 3.4) and the mean age was 30.75±18.1 (median, 26). The age group 15 to 29 years had most RTIs (41%) and most patients (50%) had elementary and intermediate education followed by those who were illiterate (22%). The majority, 59% of the patients, reported that they did not have any type of insurance (Table 1).

**Table 1:** Socio-demographic and injury characteristics of RTI victims included in INTRD, 2000-2004

**Characteristics**	**N (%)**	**Characteristics**	**N (%)**
**Sex:**		**Died in hospital:**	
Male	6479 (77.5)	Yes	402 (4.9)
Female	1876 (22.4)	No	7844 (95.1)
**Age:**		**Road user type:**	
0–4	178 (2.1)	Pedestrian	3422 (41)
5–14	1126 (13.5)	Car occupant	1639 (19.7)
15–29	3462 (41.4)	Motorcyclist	2847 (34.1)
30–44	1747 (20.9)	Bicycle rider	316 (3.8)
45–59	913 (10.9)	Others	132 (1.6)
60+	897 (10.7)	**Transportation mode:**	
**Education level:**		EMS ambulances	1049 (12.7)
Illiterate	1770 (21.5)	Other ambulances	1972 (23.7)
Elementary/Intermediate	4085 (49.6)	Other vehicles	5272 (63.4)
		Unspecified	20 (0.24)
High school	2001 (24.3)	**ISS**	
University	373 (4.3)	1–7	4004 (52.9)
**Occupation type:**		8–12	2105 (27.8)
Retired	230 (2.8)	13+	1457 (19.3)
Armed forces	184 (2.2)	**GCS**	
White-collar worker	942 (11.3)	3–8	521 (6.2)
Blue-collar worker	2192 (26.3)	9–12	444 (5.3)
Farmer	399 (4.8)	13–15	7386 (88.4)
Others*	4255 (51.1)	**Safety helmet use:**	
Unspecified	127 (1.5)	Yes	152 (5.4)
**Insurance status:**		No	2678 (94.6)
Yes	3216 (41.1)	**Seat belt use:**	
No	4601 (58.9)	Yes	87 (5.4)
		No	1533 (94.6)

RTI: Road traffic injury, INTRD: Iranian National Trauma Registry Database,

ISS: Injury Severity Score, GCS: Glasgow Coma Scale,

*Children younger than school age, students, unemployed persons, etc.

Most of the victims were pedestrians (41%). Only 5% of the car occupants had used seat-belts and the same low usage was shown for safety helmets. Five percent of the victims died in hospital. Moreover, the data showed that as many as 63% of the patients were transported to the hospital by means of transportation other than ambulance, and only 13% were transported by EMS ambulances (Table 1). The average duration of transportation to hospital by an EMS ambulance, another ambulance or by other means were 2:46 (median 1:15), 6:31 (median 3:56) and 3:40 (median 1:00) hours, respectively.

Majority of the patients (53%) had an ISS of 1–7 and GCS of 88% of patients was 13–15 in the emergency department. The mean (median) ISS and GCS scores were 10 (5) and 14 (15), respectively (Table 2). Males had higher ISS than females. Mean ISS was higher among car occupants and pedestrians followed by other road users and motorcycle riders.

**Table 2:** Total hospital charges, and LOS for RTI victims included in INTRD, 2000-2004

**Characteristics**	
**Total hospital charges (IRR)**	
Mean	1,115,819
SD	1,831,647
Median	558,680
Total range	0-46,131,061*
**LOS**	
Mean	6.8
SD	8
Median	4
Total range	1-105
**ISS**	
Mean	9.7
SD	12
Median	5
Total range	1-75
**GCS**	
Mean	14
SD	2.6
Median	15
Total range	3-15

RTI: Road traffic injury, INTRD: Iranian National Trauma Registry.

Database, LOS: length of stay, ISS: Injury Severity Score,

GCS: Glasgow Coma Scale, IRR: Iranian Rials.

(One US Dollar in 2004 was on average equal to 8,719 IRR),

* Patients with 0 charges (74 patients) are patients that their charges.

Were waived by the hospitals by different reasons. These patients.

Were excluded from the univariable and multivariable models.

### Total hospital charges and LOS

The mean total hospital charges for the patients were 1,115,819 IRR (SD=1,831,647 IRR, US$128± US$210). It ranged from 0 to 46,131,061 IRR (US$ 0- US$ 5,291). The mean LOS for the patients was 6.8 days (SD=8 days). LOS ranged from 1 day to 105 days, with a median of 5 days (Table 2).

Table 3 presents mean and median total hospital charges and LOS according to socio-demographic and injury characteristics of patients. Male patients had longer LOS (P=0.016) and higher hospitalization charges compared to women (P=0.010). Patients with a higher level of education had shorter LOS (P<0.001) and lower hospital charges (P<0.001) compared to those with lower education levels. There were no significant differences in LOS and hospital charges between different age groups. Patients with insurance stayed longer (P<0.001) and were charged more per hospitalization (P<0.001) compared to patients without any type of insurance. LOS and total hospital charges differed according to the occupation of the patients (P<0.001 for both LOS and total charges). Blue-collar and farmers had longer LOS compare with other occupational groups and retired had higher hospital charges than other groups. The differences in LOS and hospital charges for different road users were significant (P<0.001 for both LOS and total charges). Bicycle riders had the shortest LOS and the lowest hospital charges compared to other road users. There were significant differences in LOS and hospital charges between different ISS and GCS levels (P<0.001 for both LOS and total charges), indicating, as would be expected, that LOS and hospital charges increased by increasing injury severity. Patients who died in hospital had shorter LOS (P<0.001) and higher total hospital charges (P<0.001) than patients who survived.

**Table 3:** Mean and median total hospital charges and length of stay (LOS) based on socio-demographic and injury characteristics of road traffic victims included in the INTRD, 2000–2004

**Characteristics**		**LOS**		**Total hospital charges (IRR)**	
		**Mean**	**Median**	**Mean**	**Median**
Sex *	Female	6.0	5.0	966,029	440,255
	Male	6.6	5.0	1,159,487	600,625
Age	<= 4	6.1	3.0	788,973	374,320
	5 - 14	6.4	5.0	933,505	467,540
	15 - 29	6.4	5.0	1,130,350	612,229
	30 - 44	6.6	5.0	1,152,872	551,460
	45 - 59	6.3	5.0	1,086,289	535,779
	60+	6.8	5.0	1,336,937	641,535
Occupation *	Retired	6.3	5.0	1,511,020	540,710
	Armed forces	6.0	5.0	1,098,797	515,349
	White-collar worker	6.1	5.0	1,083,475	499,654
	Blue-collar worker	7.2	5.0	1,256,809	792,742
	Farmer	7.5	7.0	1,299,259	704,607
	Others	6.3	5.0	1,020,330	486,000
Medical insurance*	No	6.0	5.0	1,057,233	484,902
	Yes	7.0	5.0	1,168,149	654,635
Road user type*	Pedestrian	6.6	5.0	1,127,656	523,191
	Car occupant	6.1	5.0	1,036,314	466,085
	Motorcyclist	6.6	5.0	1,174,484	684,590
	Bicycle rider	5.4	5.0	837,929	434,853
	Others	7.2	5.0	1,177,953	654,091
ISS*	1-7	5.6	3.0	788,122	383,042
	8-12	8.0	7.0	1,487,664	1,022,613
	13+	7.2	5.0	1,635,498	831,546
GCS*	3-8	6.3	3.0	2,226,453	812,361
	9-12	8.1	7.0	1,647,298	911,787
	13-15	6.4	5.0	1,020,593	534,538
Died in hospital*	No	6.6	5.0	1,098,766	565,068
	Yes	3.8	1.0	1,464,993	274,938
Use of seat belt*†	No	6.0	5.0	1,012,010	455,587
	Yes	6.1	5.0	883,428	728,085
Use of helmet	No	6.7	5.0	1,182,108	686,574
	Yes	5.8	5.0	944,666	585,733

INTRD: Iranian National Trauma Registry Database, ISS: Injury Severity Score, GCS: Glasgow Coma Scale.

* Significant at P<0.05.

† Only significant for total hospital charges.

Multivariable analysis of total hospital charges (Table 4) showed that being at older age, having higher ISS or lower GCS, being a bicycle rider and longer LOS were associated with higher hospital charges. At the same time, being pedestrian or being car occupant, compared with bicycle riders, was associated with lower hospital charges.

**Table 4:** Multivariable analysis of total hospital charges for RTI victims included in INTRD, 2000-2004

Total charges*		**β**	**P**	**95% CI**		**β**	**P**	**95% CI**	
	LOS	0.08	0.00	0.078	0.082	0.081	0.00	0.079	0.083
Sex	Female	2.85	1.00	−0.03	0.03				
	Male**	0							
Age groups	<= 4	−0.115	0.00	−0.19	−0.04	−0.113	0.00	−0.184	−0.042
	7-14	−0.093	0.00	−0.143	−0.044	−0.082	0.00	−0.123	−0.042
	15 - 29	−0.007	0.77	−0.053	0.04	−0.012	0.51	−0.047	0.023
	30 - 44	−0.046	0.06	−0.093	0.001	−0.041	0.03	−0.078	−0.003
	45 - 59	−0.026	0.28	−0.074	0.022	−0.021	0.33	−0.064	0.021
	60+**	0				0			
Education	Illiterate	0.066	0.03	0.007	0.126				
	Elementary/Intermediate	0.088	0.00	0.035	0.141				
	High school	0.05	0.07	−0.003	0.102				
	University**	0							
Occupation	Retired	−0.023	0.51	−0.092	0.045				
	Armed forces	−0.018	0.62	−0.088	0.052				
	White collar worker	−0.008	0.67	−0.047	0.03				
	Blue collar worker	0.021	0.21	−0.012	0.054				
	Farmer	0.022	0.50	−0.042	0.087				
	Others**	0							
Road user type†	Pedestrian	−0.077	0.01	−0.135	−0.02	−0.077	0.01	−0.131	−0.022
	Car occupant	−0.07	0.02	−0.131	−0.009	−0.076	0.01	−0.133	−0.019
	Motorcyclist	−0.039	0.20	−0.098	0.02	−0.038	0.19	−0.094	0.018
	Bicycle rider**	0				0			
Medical insurance	No	0.018	0.09	−0.003	0.04				
	Yes**	0							
ISS	1-7	−0.14	0.00	−0.173	−0.107	−0.134	0.00	−0.165	−0.104
	8-12	0.021	0.24	−0.014	0.056	0.017	0.31	−0.015	0.049
	13+ **	0				0			
GCS	3-8	0.117	0.00	0.048	0.185	0.059	0.02	0.01	0.108
	9-12	0.098	0.00	0.045	0.15	0.07	0.00	0.022	0.118
	13-15**	0				0			
Died in hospital	No	0.063	0.10	−0.012	0.137				
	Yes**	0							

RTI: Road traffic injury, INTRD: Iranian National Trauma Registry Database, LOS: length of stay, ISS: Injury Severity Score, GCS: Glasgow Coma Scale.

*Model fitting information (final model): Deviance/chi-Square = 1196.88 (with d.f. of 6978 and P = 0.172).

**Reference group.

†The group “others” in road user type were not included in the analysis.

Multivariable analysis of LOS (Table 5) showed that being male, having lower education level, having a medical insurance, being a blue-collar, being pedestrian or motorcyclist, having higher ISS score or having lower GCS score were associated with longer LOS. In-hospital death and being retired were associated with shorter LOS (Table 4).

**Table 5:** Multivariable analysis of LOS for RTI victims included in INTRD, 2000-2004

LOS*		**β**	**P**	**95% CI**		**β**	**P**	**95% CI**	
Sex	Female	−0.058	0.00	−0.088	−0.027	−0.052	0.00	−0.081	−0.022
	Male**	0				0			
Age groups	<= 4	−0.056	0.15	−0.133	0.021				
	5-14	−0.017	0.52	−0.067	0.034				
	15 - 29	0.005	0.82	−0.042	0.053				
	30 - 44	0.018	0.46	−0.03	0.066				
	45 - 59	−0.001	0.98	−0.05	0.048				
	60+**	0							
Education	Illiterate	0.157	0.00	0.097	0.218	0.141	0.00	0.085	0.197
	Elementary/Intermediate	0.129	0.00	0.074	0.183	0.121	0.00	0.069	0.174
	High school	0.057	0.04	0.003	0.111	0.056	0.04	0.002	0.11
	University**	0				0			
Occupation	Retired	−0.091	0.01	−0.161	−0.021	−0.082	0.01	−0.145	−0.02
	Armed forces	−0.045	0.22	−0.117	0.026	−0.038	0.30	−0.108	0.033
	White collar worker	−0.031	0.12	−0.07	0.008	−0.02	0.28	−0.056	0.016
	Blue collar worker	0.031	0.07	−0.002	0.065	0.046	0.00	0.017	0.076
	Farmer	0.021	0.53	−0.045	0.087	0.036	0.27	−0.028	0.099
	Others**	0				0			
Road user type†	Pedestrian	0.096	0.00	0.037	0.155	0.1	0.00	0.041	0.158
	Car occupant	0.044	0.16	−0.018	0.107	0.048	0.12	−0.013	0.11
	Motorcyclist	0.079	0.01	0.019	0.139	0.083	0.01	0.024	0.143
	Bicycle rider**	0				0			
Medical insurance	No	−0.108	0.00	−0.13	−0.086	−0.109	0.00	−0.13	−0.087
	Yes**	0				0			
ISS	1-7	−0.194	0.00	−0.228	−0.16	−0.196	0.00	−0.23	−0.162
	8-12	0.022	0.23	−0.014	0.058	0.022	0.23	−0.014	0.058
	13+**	0				0			
GCS	3-8	0.151	0.00	0.082	0.22	0.151	0.00	0.082	0.219
	9-12	0.132	0.00	0.078	0.186	0.133	0.00	0.079	0.186
	13-15**	0				0			
Died in hospital	No	0.603	0.00	0.529	0.676	0.604	0.00	0.531	0.677
	Yes**	0				0			

RTI: Road traffic injury, INTRD: Iranian National Trauma Registry Database, LOS: length of stay, ISS: Injury Severity Score, GCS: Glasgow Coma Scale.

*Model fitting information (final model): Deviance/chi-Square = 1154.949 (with d.f. of 6265 and P = 0.184).

**Reference group.

† The group “others” in road user type were not included in the analysis.

### Use of safety equipment (seat-belt/helmet)

Even though very few patients reported use of safety equipment, we conducted an analysis to compare death rates, mean LOS and total hospital charges among patients who used seat-belts or helmets with patients who did not use them at the time of the collision. The analysis showed that in-hospital death for patients who used a seat-belt was 3.4% (three patients) compared to 6% (92 patients) for patients who did not use a seat-belt, however this difference failed to attain statistical significance (p=0.480). None of the injured patients who used safety helmets died in hospital compared to 3% (81 patients) of those who did not use a helmet. This difference was statistically significant (p=0.021).

The results indicate that the difference in hospital charges and LOS between the patients who did not use safety helmets and the patients who used helmets was not statistically significant (p=0.089 and p=0.154). The same results was obtained for the patients who used a seat-belt and the patients who did not use a seat-belt (p=0.126 and p=0.223 for LOS and hospital charges, respectively) (Table 3).

## Discussion

To our knowledge, this is the first national study and among few studies in the literature to analyze the association between socio-demographic factors, injury characteristics, health outcome and hospital charges and LOS among patients with RTI. The findings showed that hospital charges and LOS associated with RTIs varied by age, gender, socio-economic status, injury characteristics, health outcome of patients, and type of road users.

Although the average hospital charges in this study were lower compared to reports from HICs [6-9,32], the increasing number of RTIs and deaths in Iran, impose a huge economic burden on the Iranian society. During the years 2000 to 2004, nearly one million people were injured and more than 100,000 died due to traffic crashes in Iran [3].

Local studies conducted in hospitals in Tehran city have reported LOS ranging from 5 to 7.8 for trauma patients in general [14,33-35], which is in line with the average LOS for RTI reported in this study. It is difficult to compare LOS between countries, due to variations in the organization of trauma care and differences in injury patterns and a comparison of our results with previous studies show conflicting results. The average LOS for RTI victims in the current study was similar to the results of one study done by Odero et al., measuring health care utilization among all types of injuries in one hospital in Indiana, USA [9]. On the other hand, the mean LOS in the current study was higher than in some other studies done in HICs [6-8,32] and also in other LMICs [36]. In contrast, the average LOS for RTIs in the current study was lower than the average LOS for RTIs reported in Trinidad and Tobago [37], Kenya [38], Spain [39], New Zealand [40] and Greece [41]. The mean total hospital charges, on the other hand, were much lower in the current study compared to studies conducted in HICs [6-9,32]. Some part of the lower hospital charges, compared with HICs, can be explained by the subsidized health care system and also the general low service costs in Iran.

The findings showed that hospital charges and LOS varied with age, gender, socio-economic status, injury characteristics and health outcome of the patients. Consistent with previous knowledge [11,33,34,42], RTI victims in the current study were predominantly male (male–female ratio 3.4), mainly in productive age and from lower socioeconomic groups (both in terms of education and occupation). Men had longer LOS than women. The findings also showed that LOS decreased with increasing level of education. This result should be treated with caution since only 4% of the patients had university and higher level education. Shorter LOS in this group of patient might be explained partly by faster recovery due to self-care and better therapeutic compliance.

In line with other studies [6,7,10], patients with more severe injuries (higher ISS or lower GCS) had longer LOS and higher total hospital charges. LOS was, not surprisingly, a significant predictor of higher total hospital charges. This corroborates with other studies on the same topic in HICs [6,7]. The findings also showed that being a blue-collar worker was associated with longer LOS. Based on the information in this study and previous studies in Iran [14,33-35], the severity of injury was higher among blue-collar workers (often characterized as lower socio-economic groups, and mainly vulnerable road users, such as motorcyclists or pedestrian, in the context of Iran) compared to white-collar and other occupation groups. Different pattern of injury among blue-collar workers compared with other occupation groups might contribute to longer hospitalization periods in this group.

An important finding of the current study was the low coverage of health insurances (more than half of the patients did not have any type of health insurance) and the significant effect of having insurance on hospital utilization. The findings showed that insured patients stayed longer in hospital compared to uninsured, after controlling with socio-demographic and injury-related factors. The findings support the conclusion that lack of health insurance coverage could be a barrier preventing or limiting access to care, although the limitations of the data do not allow conclusions as to whether those covered by health insurance had better health outcomes or have access to better quality care than those not covered. Another explanation for this result can be supplier-induced demand in hospital sector in the country. Similar results were found in two studies in the US, demonstrating that uninsured or self-paying children were charged less and had shorter LOS than children covered by Medicaid [6,7]. On the other hand, a study conducted in Guadalajara, Mexico, indicated lower hospital costs for insured patients [43]. Insurance status has also been shown to be an important factor influencing utilization of health care services in general in Iran [22]. A new law was approved in Iran in 2008 (after the study period of the current study), which meant all hospital care for RTIs victims became free of charge. The effect of the new law has not been evaluated yet and further studies are needed to investigate the effect of the new law on hospital charges and LOS (and in general, resource burden) among RTI victims and also the effect of insurance on other types of traumas.

Studies have shown that patients who died during hospitalization incurred significantly higher hospital charges (mainly due to utilizing expensive intensive care during their hospitalization) but shorter LOS compared with patients who survived their injuries [6,7]. The findings from the current study indicated that the patients who died in hospital had shorter LOS compared to those survived and there was no significant difference in total hospital charges between two groups, after controlling for other factors in the multivariable model.

The findings also showed that total hospital charges and LOS varied based on type of road users. Compared with other road users, being a pedestrian was associated with lower hospital charges but longer LOS. Longer LOS for pedestrians has also been reported in previous studies [40,44]. The pattern of injury sustained by pedestrians (including head, spinal and lower extremity injuries), compared with other road users, could, to some extent, explain their longer hospital stay. Bicycle riders had higher hospital charges (but shorter LOS compared with other road users). This could be explained by higher risk of head injuries among cyclists resulting in expensive diagnostic and therapeutic procedures.

The findings indicated that use of safety equipment among the patients was very low and it did not have a significant effect on hospital charges and LOS. A new law making compulsory the use of seat-belts (for drivers and front seat passengers) and motorcycle helmets was introduced in 2001 in Iran, but based on the findings of the current study and a few other studies [13,14,33,35], the use of helmets and seatbelts seem to be exceptionally low. However, figures from recently published studies have shown an increase in the rate of seat-belt use among car occupants [45,46]. Research show that increased enforcement is one of the most effective methods to increase usage of safety equipment [1]. Since 2005 several interventions, including law enforcement of seat-belt and helmet use, have been implemented by the Traffic Police in Iran [47], however the effect of these intervention on usage of safety equipment has to be studied further

The positive effect of safety helmets and seat-belts on reducing fatal injuries is well-documented [1]. The findings of the current study that none of the patients who had used safety helmets died in hospital compared to 3% of those who had not used helmets and this difference was statistically significant. Previous studies have shown that not wearing a safety helmet or seat-belt is associated with higher hospital charges and longer LOS [16-19]. However, the results of the current study showed no significant differences in hospital charges and LOS among the patients, who did not use safety helmets and seat-belts and those who used. These findings could be explained by the small number of patients who had used safety equipment in the country.

Another important finding of this study was that the majority of patients were transported to the hospitals by means of transport other than ambulances. Low usage of EMS ambulances has also been reported in other studies in Iran [14,34,48]. In addition, the average pre-hospital transportation time reported in the current study was longer than the reported time in previous studies in urban areas of Iran [34,49,50]. Two out of three studies [49,50] which have investigating pre-hospital transportation time in Iran have measured only EMS ambulances transportation time (with average 10 and 18 minutes, recorded by EMS personnel), and one study [34] reported the average time for all transportation means (2 hours, reported by the patients). The transportation time in the current study was based on self-reports and it might have led to an over-estimation of the time. Moreover, since most patients admitted to the hospital in this study and previous studies in Iran [33,34] had moderate injury severity (low ISS and high GCS), it can be concluded that many severely-injured patients die before reaching the hospital due to more severe crashes, long pre-hospital transportation time and, insufficient pre-hospital trauma care facilities [13,51,52].

INTRD is the largest trauma registry database in Iran, but it has some limitations that may affect the findings of this study. First, the database is hospital-based and might not be representative of the pattern of RTI in general. Second, the database excluded the patients who stayed at the hospital less than 24 hours. These patients may have received care at the ED and have been discharged or died in the ED (or before reaching the hospital) because of the severity of their injuries. Excluding these patients from the database may affect the results of the study by underestimating LOS and hospital charges and in general the burden of RTI. Third, total hospital charges are billed charges and do not reflect actual payments nor true hospital costs due to factors such as government subsidies to hospital services and medicines, discounts and exemptions given to some patients by the hospitals and also not including all out-of-pocket payments by the patients. Moreover, the non-medical direct costs such as transportation costs and the indirect costs related to lost productivity, as well as the costs associated with the pain and suffering by victims and relatives are not included. Measuring these costs, although important, was beyond the scope of this study. Fourth, information such as pre-hospital transportation time and use of safety equipment (safety helmet/seat belt) are self-reported. Therefore these two variables could be over-estimated, the first one because of recall bias and the second one based on false claims (to avoid legal consequences). Moreover, in cases when the patient was too ill to report this information, it was supplied by the accompanying person, which may have led to misreporting.

Despite these limitations, the findings demonstrate a significant morbidity and resource burden associated with RTIs. By describing epidemiology, hospital charges and LOS based on different socio-demographic groups and based on outcome and injury-related characteristics of patients, the findings of this study can be used for developing interventions to improve quality of care and outcome of the patients and also help to design targeted prevention measures. Primary prevention is the preferred means of reducing the impact of RTIs on the health sector, families, and society. Use of evidence-based interventions such as increasing use of safety equipment through law enforcement and public education can be enhanced in the context of the study in order to decrease traffic-related mortality and morbidities.

## Conclusion

The study demonstrated that the hospital charges and LOS associated with RTI varied by age, gender, socio-economic status, injury characteristics and health outcome of the patients. This study showed that a majority of the patients were not insured and these patients have lower LOS than insured patients. Other important findings that need to be addressed in future research are the low usage of safety equipment and of EMS ambulances. The results of the study provide information that can be of importance in the planning and design of road traffic injury control strategies.

## Endnote

^a^ ICD-10 codes related to RTIs (fatal and nonfatal) included in the database: V02-V04 (0.1, 0.9), V09 (0.2, 0.3, 0.9), V12-V14 (0.3-0.9), V19.4-V19.6, V20-V28 (0.3-0.9), V29-V79 (0.4-0.9), V80.3-V80.5, V81.1, V82.1, V83-V86 (0–0.3), V87.0-V87.8, V89.2, V89.9.

## Abbreviations

RTIs: Road traffic injuries; LMICs: Low- and middle-income countries; GNP: Gross national product; HIC: High income countries; DALYs: Disability adjusted life years; EMS: Emergency medical services; STSRC: Sina Trauma and Surgery Research Center; INTRD: Iranian National Trauma Registry Database; ICD-10: International Classification of Diseases, 10th revision; GCS: Glasgow Coma Scale; ISS: Injury Severity Score; LOS: length of stay; IRR: Iranian Rial.

## Competing interests

The authors declare that they have no competing interests.

## Authors’ contributions

HHB was involved in the study conception and design, analysis, revision, editing and manuscript writing. SS contributed to study design, analysis, result interpretation and critical review of the manuscript. MH and LB participated to the study design, results interpretation, and finalization of the manuscript. MHY contributed to the results interpretation and finalization of the manuscript. All authors read and approved the final manuscript.

## Pre-publication history

The pre-publication history for this paper can be accessed here:

http://www.biomedcentral.com/1472-6963/13/281/prepub

## References

[B1] Peden M Scurfield R Sleet D Mohan D Hyder AA Jarawan E Mathers C World report on road traffic injury prevention 2004 Geneva: World Health Organization

[B2] Bhalla K Shahraz S Naghavi M Bartels D Murray C Road Traffic Injuries in Iran 2008 Cambridge MA: Harvard University Initiative for Global Health, Road Traffic Injury Health Metrics Group

[B3] Rahimi-Movaghar V Zarei MR Saadat S Rasouli MR Nouri M Road traffic crashes in Iran from 1997 to 2007 Int J Inj Contr Saf Promot 2009 16 3 179 181 10.1080/17457300903024277 19941217

[B4] Naghavi M Jafari N Alaeddini F Akbari ME Injury Epidemiology caused by external cause of injury in the Islamic Republic of Iran 2005 Tehran, Iran: Ministry of Health and Medical Education

[B5] World Health Organization Eastern Mediterranean status report on road safety: call for action 2010 Cairo, Egypt: World Health Organization. Regional Office for the Eastern Mediterranean

[B6] Gardner R Smith GA Chany AM Fernandez SA McKenzie LB Factors associated with hospital length of stay and hospital charges of motor vehicle crash related hospitalizations among children in the United States Arch Pediatr Adolesc Med 2007 161 9 889 895 10.1001/archpedi.161.9.889 17768290

[B7] Conner KA Williams LE McKenzie LB Shields BJ Fernandez SA Smith GA Pediatric pedestrian injuries and associated hospital resource utilization in the United States J Trauma 2003 68 6 1406 1412 20093987 10.1097/TA.0b013e3181b28b05

[B8] Shah S Sinclair SA Smith GA Xiang H Pediatric hospitalizations for bicycle-related injuries Inj Prev 2007 13 5 316 321 10.1136/ip.2007.016048 17916888 PMC2610610

[B9] Odero WW Tierney WM Einterz RM Mungai S Using an electronic medical record system to describe injury epidemiology and health care utilization at an inner-city hospital in Indiana Inj Control Saf Promot 2004 11 4 269 279 10.1080/156609704/233/289616 15903162

[B10] Christensen MC Nielsen TG Ridley S Lecky FE Morris S Outcomes and costs of penetrating trauma injury in England and Wales Injury 2008 39 9 1013 1025 10.1016/j.injury.2008.01.012 18417132

[B11] Mashreky SR Rahman A Khan TF Faruque M Svanstrom L Rahman F Hospital burden of road traffic injury: major concern in primary and secondary level hospitals in Bangladesh Public Health 2010 124 4 185 189 10.1016/j.puhe.2010.01.004 20381100

[B12] Hijar M Arredondo A Carrillo C Solorzano L Road traffic injuries in an urban area in Mexico. An epidemiological and cost analysis Accid Anal Prev 2004 36 1 37 42 10.1016/S0001-4575(02)00112-4 14572825

[B13] Roudsari BS Sharzei K Zargar M Sex and age distribution in transport-related injuries in Tehran Accid Anal Prev 2004 36 3 391 398 10.1016/S0001-4575(03)00032-0 15003584

[B14] Zargar M Sayyar Roudsari B Shadman M Kaviani A Tarighi P Pediatric transport related injuries in Tehran: the necessity of implementation of injury prevention protocols Injury 2003 34 11 820 824 10.1016/S0020-1383(02)00378-9 14580813

[B15] Sanaei-Zadeh H Vahabi R Nazparvar B Amoei M An epidemiological study and determination of causes of traffic accident-related deaths in Tehran, Iran (during 2000–2001) J Clin Forensic Med 2002 9 2 74 77 10.1054/jcfm.2002.0547 16083690

[B16] Hundley JC Kilgo PD Miller PR Chang MC Hensberry RA Meredith JW Hoth JJ Non-helmeted motorcyclists: a burden to society? A study using the National Trauma Data Bank J Trauma 2004 57 5 944 949 10.1097/01.TA.0000149497.20065.F4 15580015

[B17] Eastridge BJ Shafi S Minei JP Culica D McConnel C Gentilello L Economic impact of motorcycle helmets: from impact to discharge J Trauma 2006 60 5 978 983 10.1097/01.ta.0000215582.86115.01 16688058

[B18] Allen S Zhu S Sauter C Layde P Hargarten S A comprehensive statewide analysis of seatbelt non-use with injury and hospital admissions: new data, old problem Acad Emerg Med 2006 13 4 427 434 10.1111/j.1553-2712.2006.tb00321.x 16531597

[B19] Kerwin AJ Griffen MM Tepas JJ 3rd Schinco MA Pieper P Devin T Frykberg ER The burden of noncompliance with seat belt use on a trauma center J Trauma 2006 60 3 489 492 10.1097/01.ta.0000204030.85070.ff 16531844

[B20] World Health Organization Country profile of Iran 2008 Regional Office for the Eastern Mediterranean Available at: http://www.emro.who.int/countries/irn/. (Accessed 20 Sep 2011)

[B21] Statistical Center of Iran Iran Statistical year book 2010 Statistical Center of Iran Available at: http://amar.sci.org.ir/index_e.aspx. (Accessed 20 Sep 2011)

[B22] Hosseinpoor AR Naghavi M Alavian SM Speybroeck N Jamshidi H Vega J Determinants of seeking needed outpatient care in Iran: results from a national health services utilization survey Arch Iran Med 2007 10 4 439 445 17903047

[B23] Mehrdad R Health System in Iran The Japan Medical Association Journal (JMAJ) 2009 52 1 69 73

[B24] World Bank Islamic Republic of Iran, Health Sector Review, Volume II: Background Sections 2007 Washington DC: The World Bank Group, Human Development Sector, Middle East and North Africa

[B25] Ibrahimipour H Maleki MR Brown R Gohari M Karimi I Dehnavieh R A qualitative study of the difficulties in reaching sustainable universal health insurance coverage in Iran Health policy and planning 2011 26 6 485 495 10.1093/heapol/czq084 21303879

[B26] Haghparast-Bidgoli H Khankeh H Johansson E Yarmohammadian MH Hasselberg M Exploring the provision of hospital trauma care for road traffic injury victims in Iran: a qualitative approach J Inj Violence Res 2013 5 1 28 37 10.5249/jivr.v5i1.195 22095003 PMC3591729

[B27] Rasouli MR Moini M Khaji A Civilian traumatic vascular injuries of the upper extremity:report of the Iranian national trauma project Ann Thorac Cardiovasc Surg 2009 15 6 389 393 20081748

[B28] Salimi J Nikoobakht MR Khaji A Epidemiology of urogenital trauma in Iran: results of the Iranian National Trauma Project Urol J 2006 3 3 171 174 17559035

[B29] Moini M Rasouli MR Khaji A Farshidfar F Heidari P Patterns of extremity traumas leading to amputation in Iran: results of Iranian National Trauma Project Chin J Traumatol 2009 12 2 77 80 19321050

[B30] Schneier AJ Shields BJ Hostetler SG Xiang H Smith GA Incidence of pediatric traumatic brain injury and associated hospital resource utilization in the United States Pediatrics 2006 118 2 483 492 10.1542/peds.2005-2588 16882799

[B31] Shields BJ Comstock RD Fernandez SA Xiang H Smith GA Healthcare resource utilization and epidemiology of pediatric burn-associated hospitalizations, United States, 2000 J Burn Care Res 2007 28 6 811 826 10.1097/BCR.0b013e3181599b51 17925649

[B32] Coben JH Steiner CA Owens P Motorcycle-related hospitalizations in the United States, 2001 Am J Prev Med 2004 27 5 355 362 10.1016/j.amepre.2004.08.002 15556734

[B33] Zargar M Modaghegh MH Rezaishiraz H Urban injuries in Tehran: demography of trauma patients and evaluation of trauma care Injury 2001 32 8 613 617 10.1016/S0020-1383(01)00029-8 11587698

[B34] Moini M Rezaishiraz H Zafarghandi MR Characteristics and outcome of injured patients treated in urban trauma centers in Iran J Trauma 2000 48 3 503 507 10.1097/00005373-200003000-00023 10744293

[B35] Zargar M Khaji A Karbakhsh M Pattern of motorcycle-related injuries in Tehran, 1999 to 2000: a study in 6 hospitals East Mediterr Health J 2006 12 1–2 81 87 17037224

[B36] Perez-Nunez R Hijar-Medina M Heredia-Pi I Jones S Silveira-Rodrigues EM Economic impact of fatal and nonfatal road traffic injuries in Belize in 2007 Rev Panam Salud Publica 2010 28 5 326 336 21308177

[B37] St Bernard G Matthews W A contemporary analysis of road traffic crashes, fatalities and injuries in Trinidad and Tobago Inj Control Saf Promot 2003 10 1–2 21 27 12772482 10.1076/icsp.10.1.21.14104

[B38] Odero W Khayesi M Heda PM Road traffic injuries in Kenya: magnitude, causes and status of intervention Inj Control Saf Promot 2003 10 1–2 53 61 12772486 10.1076/icsp.10.1.53.14103

[B39] Bastida JL Aguilar PS Gonzalez BD The economic costs of traffic accidents in Spain J Trauma 2004 56 4 883 888 10.1097/01.TA.0000069207.43004.A5 15187757

[B40] Langley JD Phillips D Marshall SW Inpatient costs of injury due to motor vehicle traffic crashes in New Zealand Accid Anal Prev 1993 25 5 585 592 10.1016/0001-4575(93)90010-T 8397661

[B41] Markogiannakis H Sanidas E Messaris E Koutentakis D Alpantaki K Kafetzakis A Tsiftsis D Motor vehicle trauma: analysis of injury profiles by road-user category Emerg Med J 2006 23 1 27 31 10.1136/emj.2004.022392 16373799 PMC2564121

[B42] Odero W Garner P Zwi A Road traffic injuries in developing countries: a comprehensive review of epidemiological studies Trop Med Int Health 1997 2 5 445 460 10.1111/j.1365-3156.1997.tb00167.x 9217700

[B43] Perez-Nunez R Avila-Burgos L Hijar-Medina M Pelcastre-Villafuerte B Celis A Salinas-Rodriguez A Economic impact of fatal and non-fatal road traffic injuries in Guadalajara Metropolitan Area and Jalisco Mexico. Inj Prev 2011 17 5 297 303 10.1136/ip.2010.027995 21486986

[B44] DasGupta R Roncal S Hill D Resource utilization by injured automobile occupants and pedestrians Aust N Z J Surg 1998 68 4 271 274 10.1111/j.1445-2197.1998.tb02080.x 9572336

[B45] Majdzadeh R Eshraghian MR Khalagi K Motevalian A Naraghi K Crash-related factors associated with the severity of road traffic injuries in Iran Int J Inj Contr Saf Promot 2011 18 3 175 180 10.1080/17457300.2010.527991 21279862

[B46] Mohammadi G The pattern of fatalities by age, seat belt usage and time of day on road accidents Int J Inj Contr Saf Promot 2009 16 1 27 33 10.1080/17457300802406963 19058047

[B47] Soori H Royanian M Zali AR Movahedinejad A Road traffic injuries in Iran: the role of interventions implemented by traffic police Traffic Inj Prev 2009 10 4 375 378 10.1080/15389580902972579 19593716

[B48] Pahlavan PS Prognosis of trauma patients admitted to an emergency department in a developing country Int J Inj Contr Saf Promot 2006 13 2 117 118 10.1080/17457300500310129 16707348

[B49] Modaghegh MH Roudsari BS Sajadehchi A Prehospital trauma care in Tehran: potential areas for improvement Prehosp Emerg Care 2002 6 2 218 223 10.1080/10903120290938580 11962571

[B50] Bigdeli M Khorasani-Zavareh D Mohammadi R Pre-hospital care time intervals among victims of road traffic injuries in Iran. A cross-sectional study BMC Public Health 2010 10 406 10.1186/1471-2458-10-406 20618970 PMC2918553

[B51] Haghparast-Bidgoli H Hasselberg M Khankeh H Khorasani-Zavareh D Johansson E Barriers and facilitators to provide effective pre-hospital trauma care for road traffic injury victims in Iran: a grounded theory approach BMC Emerg Med 2010 10 20 10.1186/1471-227X-10-20 21059243 PMC2992044

[B52] Haghparast Bidgoli H Bogg L Hasselberg M Pre-hospital trauma care resources for road traffic injuries in a middle-income country–a province based study on need and access in Iran Injury 2011 42 9 879 884 10.1016/j.injury.2010.04.024 20627291

